# Photosynthesis and respiration of the soft coral* Xenia umbellata* respond to warming but not to organic carbon eutrophication

**DOI:** 10.7717/peerj.11663

**Published:** 2021-07-27

**Authors:** Susana Marcela Simancas-Giraldo, Nan Xiang, Meghan Moger Kennedy, Rassil Nafeh, Edoardo Zelli, Christian Wild

**Affiliations:** 1 Marine Ecology Department, Universität Bremen, Bremen, Germany; 2 Helmholtz Centre for Polar and Marine Research, Alfred Wegener Institute, Bremerhaven, Germany; 3 Dipartimento di Scienze Biologiche, Geologiche ed Ambientali (BiGeA) & Centro Interdipartimentale di Ricerca per le Scienze Ambientali (CIRSA), University of Bologna, Italy

**Keywords:** Octocoral, Pulsating coral, Stress modulation, Global stressor, Local stressor, DOC

## Abstract

Eutrophication with dissolved organic carbon (DOC) as a far under-investigated stressor, and ocean warming, can strongly affect coral reefs and hard corals as major reefs ecosystem engineers. However, no previous studies have investigated the metabolic responses of soft corals to DOC eutrophication, or its interaction with ocean warming. Thus, we investigated respiration and photosynthesis response of *Xenia umbellata,* a common mixotrophic soft coral from the Indo-pacific, to (1) three levels of DOC eutrophication simulated by glucose addition over the first 21 days of experiment and (2) ocean warming scenarios where the temperature was gradually increased from 26 °C (control condition) to 32 °C over another 24 days in an aquarium experiment. We found no significant difference in response to DOC treatments and all corals survived regardless of the DOC concentrations, whilst subsequent exposure to simulated ocean warming significantly decreased gross photosynthesis by approximately 50% at 30 °C, and 65% at 32 °C, net photosynthesis by 75% at 30 °C and 79% at 32 °C, and respiration by a maximum of 75% at 30 °C; with a slight increase at 32 °C of 25%. The ratio between gross photosynthesis and respiration decreased by the end of the warming period but remained similar between controls and colonies previously exposed to DOC. Our findings suggest that soft corals may be more resistant than hard corals to DOC eutrophication and in consequence, may potentially experiment in less magnitude the negative effects of increased temperature or subsequently both stressors. The results of this study may contribute to explain the successful role of soft corals in phase shifts as reported from many coral reefs. Where predicted declines in reef ecosystems health due to increased eutrophication levels can be exacerbated by future warming.

## Introduction

Coral reefs are among the most important ecosystems on earth, providing a wide variety of ecological services of high importance for human development and survival (Connell, 1978; Hughes et al., 2003). However, these ecosystems face multiple global (Doney et al., 2009; Hughes et al., 2017) and local stressors (Jackson et al., 2001; Dubinsky & Stambler, 2011; Mora et al., 2011; Lamb et al., 2018) that threaten their functionality, biodiversity, and survival. Evidence of the negative impacts of organic eutrophication and warming as local and global stressors has been widely reported for coral reef benthic communities worldwide (Walther et al., 2002; Hughes et al., 2003; Walther, 2010; Fabricius et al., 2013). In particular, eutrophication as dissolved organic carbon (DOC), has been shown to cause a direct effect on the coral holobiont through destabilization of the relationships between symbiotic algae, microbial community, and the coral host (Szmant, 2002; Kuntz et al., 2005; Kline et al., 2006; Pogoreutz et al., 2017); while DOC can also indirectly threat coral reefs by the alteration of trophic relationships in reef communities (Haas et al., 2016). Besides, thermal stress due to global warming poses a growing threat to global reefs causing hard coral bleaching and mortality (D’Angelo & Wiedenmann, 2014; Hughes et al., 2018), as sea surface temperature has risen rapidly in the past 50 years and is expected to continue rising (Poloczanska et al., 2016; Chaidez et al., 2017; Hughes et al., 2017). Under the current business-as-usual trend, warming is likely to reach an increase of 1.5 °C between 2030 and 2052 (De Coninck et al., 2018)). Thermal stress already causes depressed coral colony growth, lower reproduction rates (Baird & Marshall, 2002), and high coral mortality (Hughes et al., 2003; Walther, 2010), where these events have become more frequent and widespread during the past few decades (Baker, Glynn & Riegl, 2008).

Previous reports have shown that there is a worldwide trend of decline in coral reefs, with the possibility to lose 60% of global reefs by 2030 (Hughes et al., 2003; Bellwood et al., 2004). However, the available evidence indicates that, at a global scale, reefs may undergo significant changes in response to climate change rather than disappear entirely (Hughes et al., 2003). Coral reef dominance will most probably change, favouring the species that are more resistant to local and global stressors in future scenarios (Alvarez-Filip et al., 2013). Moreover, under ocean warming predicted for the end of the century and water pollution, phase-shifts favouring soft corals dominance could deeply transform these ecosystems (Alvarez-Filip et al., 2013; Inoue et al., 2013; Baum et al., 2016), given the differential functionality and environmental complexity that soft corals provide (Sánchez, 2016). The current evidence shows that soft corals abundance in most regions worldwide either increased or maintained, while in contrast, hard coral cover has declined over the last decades (Stobart et al., 2005; Lenz et al., 2015). Such phase shifts, associated with changes in bottom-up dynamics, can become stable through positive feedback mechanisms (Norström & Folke, 2009). In consequence, soft corals already represent the most abundant and species-rich order of octocorals on Indo-Pacific coral reefs (Fabricius, 2011), and in some areas, their density can equal or exceed that of hard corals (Fabricius et al., 2011; Lenz et al., 2015).

Soft corals ability to thrive with relatively high abundances and diversity under different and harsh environmental conditions (Fabricius & De’ath, 2008), can be explained by their particular ecophysiological characteristics. In particular, soft corals portray mixotrophic species with high nutritional plasticity (Fabricius & Klumpp, 1995; Baker et al., 2015; Schubert, Brown & Rossi, 2016; Rossi et al., 2018) where mixotrophic corals can acquire nutrients via their symbionts’ autotrophic activity and host heterotrophy (Fabricius & Klumpp, 1995).

While there are cases where mixotrophy in hard and soft corals can be comparable (Conti-Jerpe et al., 2020), it is generally accepted that soft corals show: lower dependency on autotrophy, compared to hard corals, in both shallow and deep water (Fabricius & Klumpp, 1995; Schubert, Brown & Rossi, 2016; Pupier et al., 2019), together with high variability in the contribution of heterotrophic versus autotrophic nutrition (Schubert, Brown & Rossi, 2016). This high plasticity concerning trophic strategy results in critical physiological differences in carbon acquisition mechanisms (Fabricius & Klumpp, 1995; Wild et al., 2004; Pupier et al., 2019) and ultimate energy allocation. Driving requirements for processes such as calcification (Allemand et al., 2011), growth (Lasker et al., 2003; Sánchez et al., 2004), and reproduction, which significantly differs between both groups in terms of modes and frequencies (Kahng, 2011).

Despite their high relevance as part of the benthic communities on reefs and their high potential for resistance towards climate change stressors (Schubert, Brown & Rossi, 2016; Sánchez, 2016; Rossi et al., 2020), soft corals have been strongly neglected in comparison to hard corals, which has resulted in soft corals and their responses towards stress being little or poorly understood. For hard corals, studies suggest that elevated DOC can stimulate coral-associated microbes proliferation, increasing coral hosts vulnerability (Kuntz et al., 2005; Kline et al., 2006), while exposure to organically enriched treatments can potentially reduce the resistance and resilience of hard corals to heat stress, via impacts on coral metabolism, due to changes in photosynthetic activity or respiration rates, causing bleaching and ultimately lower survival (Fabricius et al., 2013; Pogoreutz et al., 2017; Morris et al., 2019).

From the existing literature, there is little to no evidence of soft coral responses to such stressors, and it is surprisingly unclear if soft corals physiology may exhibit similar negative responses as observed for hard corals: towards increased DOC levels (Haas, Al-Zibdah & Wild, 2009; Pogoreutz et al., 2017), or warming (Al-Sofyani & Floos, 2013; Lyndby et al., 2018); or if they portray elevated or reduced thermal tolerance when simultaneously exposed to combined stressors (Fabricius et al., 2013; Pogoreutz et al., 2017). In our most recent study on this topic by Vollstedt et al. (2020), we found evidence suggesting that DOC addition had a positive effect on the heat tolerance of the soft coral *Xenia umbellata*. However, our assessment concentrated exclusively on functional and ecological variables such as pulsation rates and growth, while there is still limited knowledge about the main physiological processes taking place in these soft corals under both stressors.

It has been thoroughly reported that external nutrient availability, together with internal nutrient metabolism, can underpin the thermal tolerance of hard corals (Morris et al., 2019). Therefore, this study addresses the question of how DOC addition, warming, or both stressors combined affect photosynthesis and respiration as proxies for the metabolic status of soft corals under stress, while complementing the ecological findings from our previous study (Vollstedt et al., 2020). Here, we aimed to resolve, (1) if DOC as simulated organic eutrophication may have a negative effect on soft corals (Kuntz et al., 2005; Kline et al., 2006; Smith et al., 2006), or if soft corals can cope with this stressor (Baum et al., 2016; McCauley & Goulet, 2019), and, (2) if simulated warming causes a negative impact on these corals, while under DOC exposure that may potentially enhance or diminish coral resistance (Pogoreutz et al., 2017).

Here, we investigated the effects of single DOC treatments and the subsequent addition of warming scenarios as a further stressor in a two-stage aquaria experiment, with a total duration of 45 days. We focused on a regression-based experimental design by testing multiple exposure levels, as previously suggested by Fabricius et al. (2013). We assessed the physiological responses of the soft coral *X. umbellata* as a model*,* a widespread species found in the Indo-Pacific and the Red Sea (Al-Sofyani & Niaz, 2007; Janes, 2013), in terms of its dark respiration rates, photosynthesis, and the P:R ratio.

## Materials & Methods

### Study species and sample preparation

Species identification was performed according to the criteria by Reinicke (1997) and following the methodologies by McFadden et al. (2019). Thus, we carried out Sanger sequencing of two mitochondrial (mtMutS, COI) and one nuclear ribosomal (28S) gene PCR amplicons. The species identity was concluded to be *Xenia umbellata* (Xiang et al., 2020 unpublished data). And further confirmed based on Halász et al. (2019) and its original source, the red sea. *Xenia umbellata* (Lamarck, 1816) is a soft coral that belongs to the order Alcyonacea in the family Xeniidae. These pumping soft corals have been previously described as mainly autotrophic, portraying a reduced gastrovascular cavity and enhanced photosynthetic activity (Schlichter, Svoboda & Kremer, 1983; Kremien et al., 2013).

Fragments of this coral were propagated from mother colonies previously kept in captivity for more than two years in advance to the start of our experiments. For this study, we included 160 propagated colonies with sizes ranging between 1 and two cm^2^. Additional colony fragments of the same species (140 colonies) were present in the experimental system but reserved for a different study. Furthermore, each colony was attached to a coral frag plug (Aqua Perfect plug/ Round one cm (AP-7004-0)) employing rubber bands and allowed to subsequently heal for at least 10 days before the start of acclimation period. These colony fragments were initially maintained under controlled conditions at a main tank in the aquaria facilities, filled with artificial seawater at a salinity of 35 ± 0.2 ppt, pH of 8.2 ± 0.01, temperature of 25.6 ± 0.6 °C (mean ± SE), and exposed to a 12:12 photoperiod at constant light intensity. Once healed, the 160 colonies colonies were subject to 11 days acclimation, and subsequently, randomly distributed into the tanks of our experimental system, allocating ten colonies per tank.

During the experiment’s total extent, the colonies were continuously monitored to assess and record their evolution over time and control for the occurrence of bleaching. For instance, we performed a swift check on the coral colonies’ survival percentages, starting with the 10 initial colonies in each tank. From there on, the colonies were continuously tracked daily through visual and direct inspection of tissue firmness, colony aspect and coloration, together with photographs taken overtime at the end of each incubation day. We accompanied these inspections with additional confirmation from our respiration rate records over time. On the other hand, we used the information collected on colony coloration and photographic confirmation as a qualitative approximation to bleaching. Observations were documented, for initial ease, as presence-absence of conspicuous signs of bleaching, understood as complete and prominent whitening of the coral colonies. We collected additional notes on paler colorations on sight and further contrasted our visual inspections to photosynthetic activity records, aiming to evaluate in detail the colonies upon the occurrence of bleaching.

### Experimental setup

Our study took place at the research facilities of the Marine Ecology group in the Center for Environmental Research and Sustainable Technology (UFT) at University of Bremen, Germany.

The experiment consisted of two phases: a first phase that included single DOC additions and a second phase that consisted of additional thermal stress treatments. The experimental essays were carried on a close aquaria system, including 16 individual glass tanks, each with a total volume of 60 L. In further detail, each of these tanks was divided into two technical parts: a 50 L front part, housing the experimental colonies, and a 10 L back part functioning as filtration tank or sump, holding a pump (EHEIM CompactOn 300 pump; EHEIM GmbH and Co. KG, Germany), a heater to manipulate and control temperature (3613 aquarium heater. 75 W 220–240 V; EHEIM GmbH and CO. KG, Germany), a skimmer (EHEIM SkimMarine 100; EHEIM GmbH and Co. KG, Germany), and an outflow providing constant water exchange between the front and the back part of the tank. Illumination was provided by LED lights (Royal Blue –matrix module and Ultra Blue White 1:1 –matrix module, WALTRON daytime^®^ LED Light, Germany), in an array of blue and white combination arrangement equally adjusted for each tank and at a constant mean intensity of 120.8 ± 10.2 µmol quanta m^−2^ s^−1^ and exposed to a 12:12 photoperiod. Moreover, the experimental tanks were filled with artificial seawater prepared with sea salt (Tropic Marin^®^ ZooMix Sea Salt) free from synthetic additives, nitrates and phosphates, and containing all trace elements found in natural seawater concentrations. The aquaria were maintained at equivalent conditions to those in the main tank with salinity of 35.4 ± 0.4 ppt, pH of 8.2 ± 0.01, and temperature of 26 ± 0.4 °C (mean ± SE) during the first 21 days, before temperature the treatments. Salinity, temperature and evaporation rates were monitored continuously three times per day and adjusted if required, while additional chemical water parameters, such as the pH, KH, Ammonium (NH_4_^+^), Nitrite (NO_2_^−^), Nitrate (NO_3_^−^) and Phosphate contents (PO_4_^3−^) were measured and adjusted manually twice per week. Dissolved O_2_ concentrations in the tanks were unaffected by the enrichment, remaining always higher than 6 mg/L. Further details on chemical parameters and their recorded values can be found summarized in Vollstedt et al. (2020).

Furthermore, 10% water exchanges were performed in the experimental tanks daily during the mornings, ensuring near to natural sea water parameters and to in order to maintain algae growth under control.

### Organic enrichment and temperature treatments

In order to assess the effects of organic eutrophication on *X. umbellata*, 12 of the experimental aquaria were randomly arranged into three DOC treatments and a control condition. Thus, the coral colonies arranged in the experimental tanks, were kept under three DOC concentration treatments: low concentration of 10 mg L^−1^, a medium concentration of 20 mg L^−1^ and a high concentration of 40 mg L^−1^. Organic enrichment was performed daily through glucose (D-Glucose) additions, while the control condition was represented by untreated aquaria (2 to 3 mg L^−1^). The colonies remained incubated in the tanks under the beforehand mentioned conditions during the whole execution of the experiment. To keep DOC concentrations constant in our treatments, the DOC contents of each tank were measured twice a day with a TOC Analyzer (TOC-L CPH/CPN PC-Controlled Model, Shimadzu, Japan). Measurements were always performed every morning and, in the afternoon, (10:00 and 17:00). Using the results from the morning measurements, the required amount of glucose to be added after consumption was calculated, and subsequently added in the afternoon from a mother solution freshly prepared every day, before the second monitoring measurement. This first phase of the experiment had a total duration of 21 days, with 10 incubation time data points collected at higher frequencies during the initial week of the experiment, and including a base line incubation before the start of the treatments.

During the second phase of the experiment, i.e., from day 21 and until day 45, warming effects on *X. umbellata* corals were assessed through three temperature treatments. Four additional tanks (procuring the same conditions as the DOC untreated controls), were prepared and included as controls for the temperature treatment. This controls, remained untreated with DOC and at a constant temperature of 26 °C until the end of the experiment.Thus, the experimental tanks were arranged as 16 aquaria, with four of them as the beforementioned controls, and 12 of them representing the low, medium and high DOC concentrations plus a DOC untreated control. The later 12 tanks, experienced the temperature treatments achieved through stepwise increases over time: 28, 30, and 32 °C. In further detail, on day 21 of the experiment a slow temperature increase of 2 °C in a matter of 5 days was performed. It was then kept stable for three days after reaching the target temperature, and physiological measurements were performed before increasing the temperature again by 2 °C more until reaching the maximum of 32 °C. Temperature treatments were selected and conducted in consideration of the IPCC report 2018, to which natural temperature changes would apply (De Coninck et al., 2018)). A total of nine incubation data points in time, plus a baseline were collected during this second phase.

### Photosynthesis and respiration rates

Physiological measurements were carried out following the established beaker incubation technique by Herndl & Velimirov (1986) and procedures described by Bednarz et al. (2012) for the same xeniid species. Net photosynthesis and respiration were measured for three organisms per treatment (i.e., 3 coral colonies per 4 experimental conditions during the first experimental phase (*n* = 12), and 3 coral colonies per 5 experimental conditions distributed in the 16 tanks for the second experimental phase, thus (*n* = 16). Our incubations were always performed in the mornings (∼2 h after LED lamps switched on in the system). As higher respiration rates in light-adapted corals relative to dark-adapted corals (Anthony & Hoegh-Guldberg, 2003; Porter et al., 1984) provide a better estimate for calculating gross photosynthesis (Bednarz et al., 2012). At the start of each measurement day, one coral colony was randomly selected from the ten organisms available in each tank and picked from it. The selected colonies were collected underwater in 151 mL glass jars to avoid air exposure. Observing they did not show signs of stress due to manipulation and remained pumping during the measurements. The initial O_2_ concentrations were measured in each jar using a salinity corrected oxygen optode (HACH HQ40d multimeter, HACH Lange GmBH and Co., Germany). To measure planktonic background metabolism, a single plug containing no coral colony was recovered from each aquarium and introduced into a glass jar following the same procedure as collected colonies. These plugs served as controls undergoing the same treatments as each of the colonies chosen. Once measured, the jars were carefully closed airtight, avoiding the presence of air bubbles and taken for incubation procedures.

Dark incubations took place in a darkened room, inside water baths conditioned in order to ensure no light penetration and constant temperature. After 2–3 h incubation, the jars where re-opened, directly measured and end time and O_2_ concentration values were recorded per each sample. While photosynthesis may exhibit a lag response during the first minutes of dark incubations, O_2_ evolution can be considered negligible under the context of 2 to 3 h incubation time (Bednarz et al., 2012). To obtain net photosynthetic activity, we performed 2–3 h of light incubations. After measuring the start O_2_ concentration for every sample, we closed airtight each glass jar and placed it back into the experimental system. We allocated each jar inside the original aquarium from which its colony (or single plug) was picked. All the jars remained in the experimental tanks to ensure constant irradiance and temperature during the incubation procedure. When the incubation time was finally over, each jar was re-opened and measured once more to record end time and O_2_ concentrations of each sample.

Furthermore, O_2_ fluxes obtained from the dark and light incubations were normalized by incubation time (hours; mg O_2_ L^−1^ h^−1^), corrected for incubation volume and normalized to coral surface area (mg O_2_ m^−2^ h^−1^), therefore obtaining gross, net photosynthesis and respiration rates as a final result from the calculations. The surface area of the colonies was calculated using the approach by Bednarz et al. (2012), where area approximation of a polyp (which we obtained through assessment of taken photographs of the individual colonies), can be multiplied by the number of polyps in each colony, and counted for each colony after finishing each incubation.

### Data analysis

All statistical analyses were performed in the computing software R version 3.0.2 (R Development Core Team, 2013), using the Lme4 package (Bates et al., 2015). To test for differences in the treatments, a Linear Mixed-effects model (LMM) analysis was used for obtaining fitting models to the data per each predictor assessed. We evaluated data normality for each physiological response using a Shapiro–Wilk test and Q-Q plots as an additional aid for visual inspection. We carried out model diagnostics as the models were being constructed, and Pearson’s residuals variance was evaluated through residual plots to confirm that fitted model assumptions were met. Models were calculated separately for each experimental phase, including the complete data for all the incubations performed in the first 10 data time points plus baseline for the first phase of the experiment and 9 data time points plus day 21 as a baseline for the second experimental phase with simulated warming. To assess model sensitivity, we evaluated further Cook’s distances and data leverage. Two data points identified as strong outliers (magnitude way larger than >1.5 times the interquartile range of the data) were removed from the analysis after outlier treatment since they prevented model convergence. Furthermore, for the LMM analysis, the factors evaluated as fixed variables included: (1) DOC concentration: four levels for the first part of the experiment and five levels for the subsequent part, including warming conditions as a stressor. (2) Temperature: three levels factor, only tested for the second part of the experiment, where warming temperatures were included as a treatment. The variable time was also included to evaluate the factors in a longitudinal assessment. The interaction between the factors DOC × time and DOC × temperature was evaluated as well. The significance of fixed effects was determined using an ANOVA type III (Zuur et al., 2009), with Satterthwaite’s method for degrees of freedom approximation (Kuznetsova, Brockhoff & Christensen, 2014). Models were considered statistically significant when found to be supported by *p*-values of *P* < 0.05. Where significant differences were observed, we performed additional Tukey tests (Supplemental Information S1 and S2), using the ‘glht’ function from the multcomp package (Hothorn, Bretz & Westfall, 2008) and the package emmeans (Lenth, 2019) for results confirmation and visual inspection.

## Results

During the first phase of the experiment, i.e., throughout the first 21 days, all the coral colonies survived regardless their exposition to the DOC concentration treatments. In contrast, by the end of the subsequent warming period, i.e., from day 21 to 45, mortality and visible stress signs were observed only for the heat-stressed controls in the absence of DOC (Vollstedt et al., 2020). For these colonies, mean survival decreased to 80.9% while 38% of the surviving ones in this condition showed size reductions, shrunk polyps, thinner stalks and depressed tentacles with compromised pinnate structures. Moreover, the colonies under warming and in the different DOC concentrations retained a less stressed and relatively healthy appearance, while their survival was of 100% until the end of the experimental term. Subtle lessening in coloration occurred in 41% of the colonies in the high DOC concentration treatment and for 26% of the colonies in the medium DOC concentration treatment. However, no conspicuous sign of bleaching, understood as complete whitening of the colonies accompanied by null photosynthetic activity, was observed for any of the treatments during the extend of the experiment.

### DOC organic enrichment

**Figure 1:**
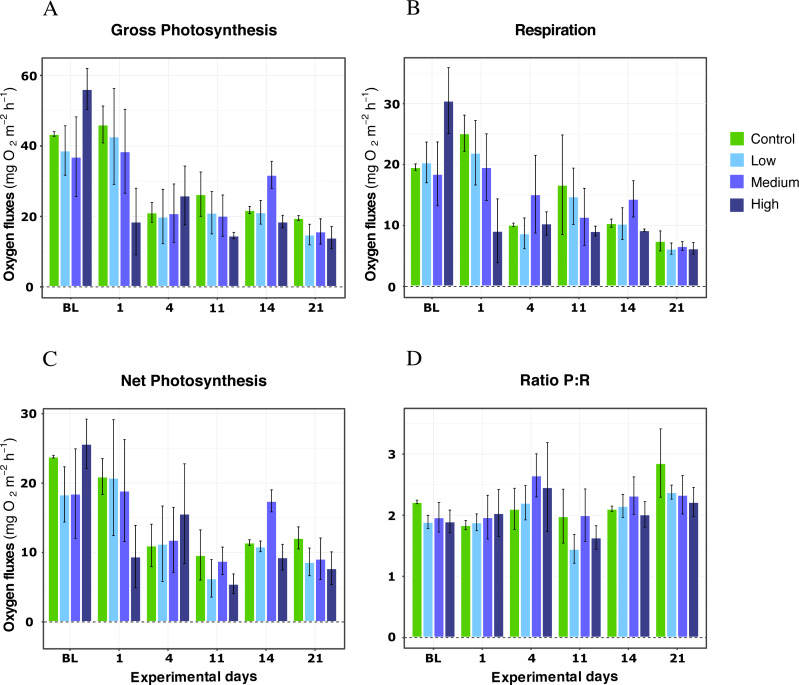
Respiration rates, net photosynthesis, gross photosynthesis (mg O_2_ m^−2^ h^−1^), and P:R ratio of *X. umbellata* under single DOC simulated organic eutrophication over time. Control condition: 2–3 mg/L (green), Low: 10 mg/L (light blue), Medium: 20 mg/L (medium blue), and High: 40 mg/L (dark blue). Bars values indicate mean ± s.e.m. for *n* = 12 (3 samples ×4 conditions).

For *X. umbellata* colonies exposed to different DOC concentrations during the first 21 days of the experiment, no impact of the DOC treatments was observed for any metric (Table 1). Thus, the *p*-values for DOC concentrations treatments were not significant for gross photosynthesis response (LMM; *F* = 1.21, *P* = 0.39; Fig. 1A), respiration (LMM; *F* = 1.06, *P* = 0.42; Fig. 1B), net photosynthesis (LMM; *F* = 1.39, *P* = 0.34; Fig. 1C), or the P:R ratio (LMM; *F* = 0.59, *P* = 0.64; Fig. 1D). The interaction between DOC treatments and time was not significant either for any of the metrics assessed (LMM; *P* > 0.05; Table 1). Only the variable time was significant (LMM; *P* < 0.05) for every metric. Net photosynthesis and respiration, and thus gross photosynthesis, showed initial higher values, which reached stability after four days of treatments exposition and remained stable until the end of the experiment’s first phase. In particular, gross photosynthesis rates reached similar mean values among treatments rounding 22 ± 7.00 mg O_2_ m^−2^ h^−1^ on average, while the P:R ratio response rounded 2.5 ± 0.3 by day 21 of the experiment, well above 1.5, a conservative threshold for net autotrophy (Wilkinson, 1983).

**Table 1:** Linear mixed-effects model results for gross and net photosynthesis, respiration rates (mg O_2_ m^−2^ h^−1^), and the P:R ratio of *X. Umbellata corals* under single DOC addition. Type III analysis of variance with Satterthwaite’s approximation method for degrees of freedom.

**Factor**		**Gross** **photosynthesis**		**Respiration**		**Net** **photosynthesis**		**P:R ratio**	
**Fixed effects**	***df***	**F**	**p**	**F**	**p**	**F**	**p**	**F**	**p**
DOC	3	1.206	0.386	1.06	0.421	1.394	0.338	0.589	0.639
Time	9	6.712	**9.20e**^−07^***	6.41	**1.464e**^−06^***	3.371	0.00186 **	2.370	0.0213*
DOC × Time	27	1.215	0.257	1.09	0.379	0.955	0.538	0.939	0.558

**Notes.**

*P*-values defined as significant at a threshold of *P* < 0.05 are highlighted in bold.

### Warming temperature treatments and DOC addition

When exposed to simulated warming scenarios, there was a significant effect of the temperature factor for every metric (LMM; *P* < 0.05; Table 2). A general decline was observed in response to the increased temperatures, together with significant differences between most pairwise temperature comparisons (Supplemental Information S1 and S2) In contrast, no significant effect was found for the DOC treatments or the interactions between DOC and Temperature, or DOC and time for any metric (LMM; *P* ≥ 0.05; Table 2). Furthermore, significant effects observed were primarily due to the factor temperature and not to the factor time, which was not significant for most metrics (LMM; *P* > 0.05; Table 2), except respiration (LMM; *F* = 4.70, *P* < 0.05; Table 2).

**Table 2:** Linear mixed-effects model results for gross and net photosynthesis, respiration rates (mg O_2_ m^−2^ h^−1^), and the P:R ratio of *X. Umbellata* corals under simulated warming and continuous DOC addition. Type III analysis of variance with Satterthwaite’s approximation method for degrees of freedom.

**Factor**		**Gross** **photosynthesis**		**Respiration**		**Net** **photosynthesis**		**P:R ratio**	
**Fixed effects**	***df***	**F**	**p**	**F**	**p**	**F**	**p**	**F**	**p**
DOC	4	0.855	0.515	0.772	0.563	1.505	0.206	2.440	0.0512.
Temperature	3	33.13	**6.3e**^−15^***	17.90	**2.35e**^−09^***	36.87	**<2e**^−16^***	11.06	**2.16e**^−06^***
Time	7	1.902	0.0774.	4.701	0.00014 ***	1.562	0.155	1.585	0.147
DOC × Temp.	12	0.551	0.876	0.947	0.504	0.705	0.743	0.924	0.526
DOC × Time	24	0.553	0.951	0.760	0.776	0.622	0.909	1.487	0.087

**Notes.**

*P*-values defined as significant at a threshold of *P* < 0.05 are highlighted in bold.

**Figure 2:**
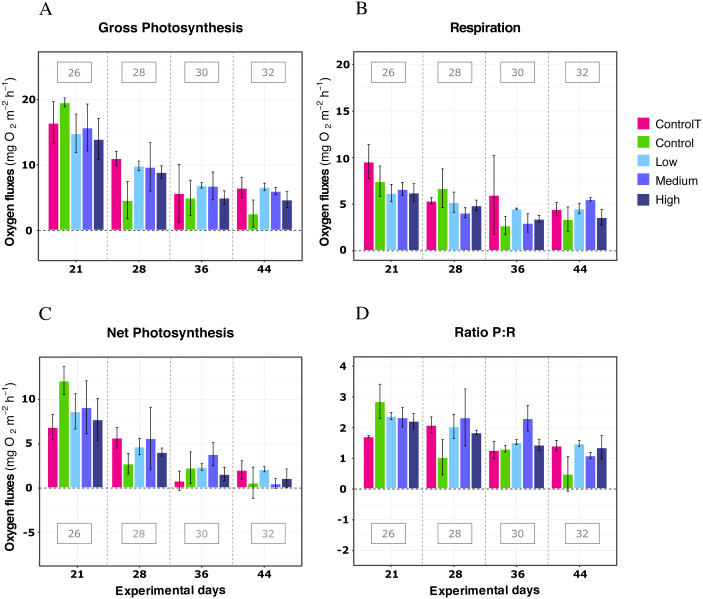
*X. umbellata* response in terms of respiration rates, net photosynthesis, gross photosynthesis (mg O_2_ m^−2^ h^−1^), and P:R ratio to warming stress and prolonged DOC addition over time. Control condition for the temperature treatment (ControlT): 2–3 mg/L DOC at constant 26 °C over time (pink), heat-stressed control (Control): 2–3 mg/L (green), Low: 10 mg/L (light blue), Medium: 20 mg/L (medium blue), and High: 40 mg/L (dark blue). Bars values indicate mean ± s.e.m. for *n* = 16 (3 samples × 4 heat stressed conditions plus 4 samples for the non-heat stressed control).

**Figure 3:**
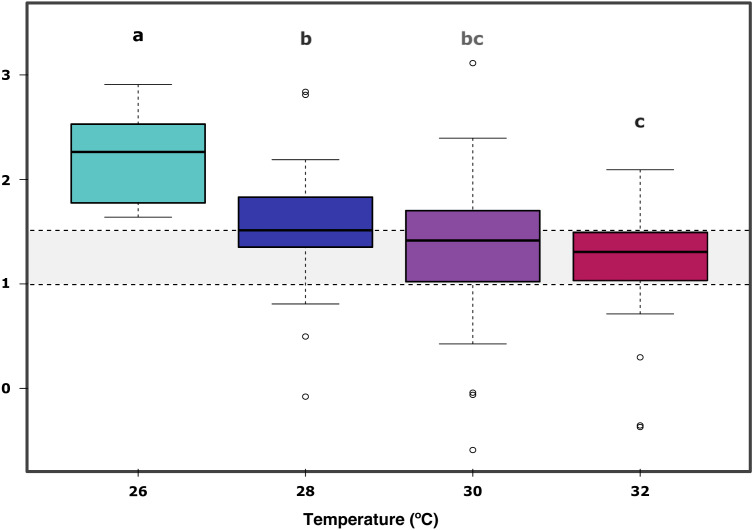
*X. umbellata* P:R ratio trend as a function of increased temperature. Grey space between dashed lines represents the zone between compensation where P:R = 1.0 and the conservative threshold for net autotrophy P:R > 1.5. Letters, together with colors indicate significant differences between temperature groups as determined by Tukey’s test (*P* < 0.05) on the P:R metric.

In further detail, gross photosynthesis (Fig. 2A) showed approximately a 50% decrease from the 26 °C to the 28 °C condition, and from 28 °C to 30 °C. At 32 °C, an overall 65% reduction was observed compared to the 26 °C condition for all DOC treatments. Besides, respiration response at 28 °C, portrayed mean values being 50% less than the recorded ones at the start of the temperature stress phase, and a further 75% reduction at 30 °C for the heat-stressed control together with the DOC treated conditions; followed by a 25% positive increase from 30 to 32 °C (Fig. 2B). Moreover, net photosynthesis (Fig. 2C) showed a substantial reduction of approximately 79% at 28 °C for the heat-stressed controls, while the DOC treatments and the temperature treatment control decreased together, in lesser magnitude. After temperature increased to 30 °C and subsequently to 32 °C, all the DOC treatments and controls alike decreased even further by approximately 75% at 30 °C and 79% at 32 °C, with respect to 26 °C. On the other hand, the P:R ratio (Fig. 2D) showed statisticaly comparable mean values across all the DOC treatment conditions and both controls, together with a steady decrease from 26 °C through 28 °C, 30 °C and at 32 °C. When contrasting temperature responses (Fig. 3), all pair comparisons were significant for the P:R ratio (Table S1.4, Fig. S2.4; *p* < 0.05), except 28 °C to 30 °C and 30 °C to 32 °C. At 28 °C coral colonies had average P:R values rounding 1.5, while at 30 °C and 32 °C most P:R values remained approximately between 1.5 and 1.0, where P:R = 1.0 defines compensation.

## Discussion

Our study of the effects of DOC eutrophication as glucose addition and simulated warming on the soft coral of *X. umbellata* shows that net and gross photosynthesis, respiration and the P:R ratio are completely insensitive to DOC organic enrichment, even under exceptionally high concentrations at both, 26 °C and increased temperatures. This contrasts with hard corals, which are negatively impacted by DOC at non-stressful temperatures and therefore, potentially impacted by DOC under heat stress. Moreover, the detrimental response of *X. umbellata* to temperature stress, however milder for these corals when compared to many other hard coral responses to thermal stress (Prada, Weil & Yoshioka, 2010; DeCarlo et al., 2017; Osman et al., 2018; Monroe et al., 2018; Ziegler et al., 2019), demonstrates that *X. umbellata* shows considerable resistance to this stressor, even though higher temperatures may negatively drive the photosynthesis and respiration response in this species. These results agree with the suggested outcome from the ecological response for *X. umbellata,* where pulsation rates did not suggest disturbances caused by DOC addition but rather under simulated warming at 30 °C and 32 °C (Vollstedt et al., 2020). However, no statistical significance was found for the interaction term between temperature and DOC for any of the response variables at any temperature step. From the physiological perspective in our present study, we found no beneficial effect of DOC on *X. umbellata* response, despite the suggested benefit of DOC on the ecological variables assessed in our previous study. Thus, there seem to be differences between the ecological and physiological metric responses of *X. umbellata* to DOC and heat stress, and ecological benefits of DOC did not appear to translate into physiological benefits. Furthermore, DOC did not increase sensitivity to warmer temperatures in this soft coral, in contrast to hypothesized expectations where warming scenarios may pose a greater threat given the ubiquitous presence of diazotrophs in most coral holobionts (Pogoreutz et al., 2017). Our results support the likelihood that the mixotrophic *X. umbellata* soft coral may be potentially able to endure challenging conditions imposed by high eutrophication better than their counterpart, the calcifying hard corals. DOC resistance may convey a considerable advantage for soft coral dominance, potentially even under future changing climate scenarios.

### Effects of single DOC addition on respiration and photosynthesis

There was no effect of single DOC addition on *X. umbellata* photosynthesis or its respiration rates, regardless of the concentrations assessed. Previous studies on the effects of organic enrichment on hard corals have extensively reported its adverse effects for many species. For instance, decreased photosynthetic activity and additional negative impacts in photosynthesis-related metrics have been described by, e.g., Pogoreutz et al. (2017), for the hard coral *Pocillopora verrucosa*, by Haas, Al-Zibdah & Wild (2009) for *Acropora sp.*, by Fabricius et al. (2013) for *Acropora millepora* and *Montipora tuberculosa* and, by Weber et al. (2012) for *Montipora peltiformis*. In particular, Pogoreutz et al. (2017); Pogoreutz et al. (2018) reported already strong damaging effects for concentrations such as 10 mg L^−1^, while the study of Kline et al. (2006) showed that DOC levels of 25 mg L^−1^, compared to our medium level concentrations (i.e., 20 mg L^−1^), caused disruptions of the balance between the hard corals and their associated microbiota. In contrast, after additional acclimatization within the first days of the experiment, photosynthesis and respiration rates of *X. umbellata* in our study were comparable to values expected for healthy untreated colonies, in agreement with previous references by Bednarz et al. (2012). Both parameters remained relatively constant until the end of the single DOC addition exposure phase, i.e., the first 21 days. Besides, no detrimental effects seemed to have occurred due to over-stimulated growth of coral mucus-associated microbes expected from organic carbon loading with glucose (Kuntz et al., 2005).

Furthermore, the absence of a DOC effect on photosynthesis in our study may indicate that the requirements for this physiological process were already being met through the available energy byproduct of regular metabolism (Comeau, Carpenter & Edmunds, 2017), while a lack of an increase in photosynthesis could suggest that the *Symbiodinium* of *X. umbellata* did not make use of the readily available carbon source. Stark increases in net and gross photosynthesis activity under high DOC concentrations have been reported to happen via heightened CO_2_ availability in *hospite* in another pumping cnidarian (Rädecker et al., 2017). However, this seemed not to be the case in our study. As an additional observation from the physiological point of view, coral calcification can be enhanced through different mechanisms. Indirectly, via increasing the photosynthetic activity of the symbionts (Goreau, 1959; Falkowski et al., 1984; Muscatine, 1990), where calcification and photosynthesis have been traditionally accepted as tightly coupled (Goreau, 1959; Falkowski et al., 1984; Muscatine, 1990; Burriesci, Raab & Pringle, 2012; Kopp et al., 2015); and also directly, via light absorption by the coral host (Cohen, Dubinsky & Erez, 2016; Eyal et al., 2019) and heterotrophic feeding (Houlbrèque & Ferrier-Pagès, 2009). Calcification processes in soft corals such as xeniids may not be as energetically costly as calcification in hard corals; this might enable the coral to have a larger energetic budget as surplus, also from photosynthesis, that may be available for resistance and other processes, e.g., pumping, growth or mucus production. Furthermore, differences in mineralized structure components, intracellular or extracellular calcification, and calcite or aragonite incorporation can influence the photosynthesis response to specific stressors (Hofmann & Bischof, 2014). Under environments in which energetic demands may exceed metabolic capacity, individuals with maintenance costs less sensitive to environmental stressors may be more likely to survive by changing the allocation of their available energy (Pan, Applebaum & Manahan, 2015).

On the other hand, the null effect of DOC on respiration could reflect multiple pathways of metabolic responses, ranging from potentially no effect to more complex scenarios involving energetic tradeoffs among critical processes that change the allocation of energy to individual functions while conserving the overall metabolic costs (Pan, Applebaum & Manahan, 2015; Comeau, Carpenter & Edmunds, 2017). Moreover, a negligible effect of sugar enrichment on respiration (and net and gross photosynthesis), due to only DOC addition contrasts with previous findings from works on other soft-bodied cnidarians and hard corals. Where glucose enrichment has been shown to stimulate respiration in the cnidarian mixotrophic model organism, *Cassiopeia* sp., (Rädecker et al., 2017), and to increase respiration rates while decreasing gross photosynthesis in the hard coral *P. verrucosa* as in Pogoreutz et al. (2017).

Regarding the P:R ratios, there was no statistical evidence of physiological dose–response relationships occurring by using multiple DOC levels of exposure. Therefore, the colonies remained healthy and autotrophic, regardless of DOC as a stressor, suggesting that until 40 g L^−1^ of DOC concentration, DOC levels were still inside the range of *X. umbellata* response optima with P:R ratios by the end of the single DOC addition period comparable to non-treated *X. umbellata* corals as reported by Bednarz et al. (2012). These findings are consistent with non-affected pulsation rates described by Vollstedt et al. (2020), regardless of DOC concentrations.

### Warming temperatures effects and its synergy with DOC

During the warming phase of our study, the temperature had a significant effect on net and gross photosynthetic activity, which showed a negative trend proportional to temperature increases. For both metrics, all pair temperature contrasts were significant (Tables S1.1 and S1.3, Figs. S2.1 and S2.3; *p* < 0.05), except 30 °C to 32 °C. Besides, the DOC treatments and the control remaining at 26 °C did vary together with the temperature treatments. Substantial decreases of photosynthetic O_2_ evolution and maximum quantum yield are known to be early responses of Symbiodinium to heat stress, following the overwhelming of photoprotective mechanisms (Jones et al., 1998). However, it is remarkable that for *X. umbellata*, positive net photosynthesis was still observed at 32 °C, although harshly reduced. These results suggest that the expected impact of elevated temperatures on *X. umbellata* may be much lesser than previously suspected (Strychar et al., 2005).

Regarding respiration, a decrease was observed through 28 °C and 30 °C, with a slight increase again from 30 °C to 32 °C (Table S1.2, Fig. S2.2; *p* <  0.05). A decrease in respiration under stress may be indicative of a reduction in metabolic costs and perhaps explicit metabolic depression (Pörtner, Langenbuch & Reipschläger, 2004). While in contrast, elevated respiration as a function of thermal stress could result from higher-performing costs, as it occurs in the case of increased respiration rates under elevated pCO_2_; caused, for example, by perturbed active cellular transport or an increasing cost of other metabolic activities (Comeau, Carpenter & Edmunds, 2017).

Furthermore, the ratio P:R trend showed a steady decrease from 26 °C through 28 °C, 30 °C and finally 32 °C, all significant when compared to 26 °C (Table S1.4, Fig. S2.4; *p* < 0.05); together with longitudinal significance of the temperature. A significant difference in the 26 °C to 28 °C steps suggests already thermal stress responses to a 2.0 °C increase in temperature. Nevertheless, the P:R ratio remained above the compensation threshold (P:R = 1.0) at 28 °C, 30 °C and 32 °C for all the temperature treatments, but not for the heat-stressed control at 32 °C, which also showed a decrease in survival. The coral colonies remained mostly autotrophic until 32 °C with P:R ratios above compensation (P:R >1.0) but below or rounding the conservative net autotrophic threshold (P:R >1.5) for 30 °C and 32 °C.Thus, *X. umbellata* ratios described here suggest strongly net autotrophic behaviour at 26 °C, moving within the heterotrophic –autotrophic continuum towards a more heterotrophic character at 30 °C and 32 °C (Wilkinson, 1983; Baker et al., 2015). The autotrophic P:R ratio observed at 26 °C can be considered higher than those of other soft corals species. However, still in the lower range compared to observations for hard corals, rounding 2.0 to 4.0 (Fabricius & Klumpp, 1995; Bednarz et al., 2012). It is as well particular that the *X. umbellata* P:R ratio is far lower than the 8.3 ratio recorded for the equally pulsating xeniid *Heteroxenia fuscescens* (Kremien et al., 2013). Nevertheless, its ability to pulsate may allow it to reach higher energetic yields than other non-pulsating corals (Kremien et al., 2013). Altogether, diversity of P:R ratios among taxa may reflect the differential potential for species-specific tolerances towards stressors, where modulation strategies and coping mechanisms may highly vary and be dependent on the particular nature of the stressor and the available energetic budget of the coral holobiont (Pan, Applebaum & Manahan, 2015) in complement to its nutritional plasticity (Schubert, Brown & Rossi, 2016; Rossi et al., 2020; Conti-Jerpe et al., 2020), associated microbial community dynamics (Neave et al., 2019), and surrounding seawater conditions.

Besides *X. umbellata* potential ability to enhance its autotrophic energy input, these soft corals have as well a non-negligible mixotrophic component. The P:R trend observed through temperature increases suggests that corals would go in the direction of shifting to heterotrophy by reaching values below compensation (Baker et al., 2015), given enough time. Traditionally, soft corals have been described to rely more on heterotrophic feeding due to their lower photosynthetic productivity when compared to hard corals, as supported by lower P:R ratios rounding 1.0 to 1.3 (Fabricius & Klumpp, 1995). Moreover, heterotrophic feeding has been shown to aid various hard coral species in sustaining a positive energy balance under thermal stress or eutrophication (Anthony & Fabricius, 2000; Borell & Bischof, 2008; Conti-Jerpe et al., 2020). However, in our study, particular heterotrophic uptake of DOC as glucose, as mechanism to endure thermal stress could not be confirmed from the physiological point of view, given the lack of statistical significance for the interaction term between DOC and temperature together with lack of evidence favouring utilization of already available carbon (no significance for the DOC factor in any metric). These findings contrast to earlier studies where *X. umbellata* has been suggested to balance carbon deficiency under inorganic nutrients enrichment by increasing DOC heterotrophic uptake (Bednarz et al., 2012). While other soft corals are known to able to uptake dissolved organic matter directly as an energy source, especially when available in excess (Schlichter, 1982). In our case, we cannot discard that *X. umbellata* could eventually use this or other resources to sustain its metabolism if its behaviour eventually shifts towards a sustained heterotrophic regime. Nevertheless, this suggestion remains to be further studied for confirmation.

As an additional remark, DOC eutrophication has been shown to induce bleaching in hard corals but did not in *X. umbellata*. For instance, sugar enrichment, without temperature and light stress, can initiate a bleaching response in the hard coral *Pocillopora verrucosa* together with gross photosynthesis decrease (Pogoreutz et al., 2017). On the other hand, thermal stress has been thoroughly reported to cause bleaching in hard corals (Weis, 2008; Santos et al., 2014; Cardini et al., 2016; Hughes, Kerry & Simpson, 2018; Ziegler et al., 2019) and soft corals as observed in, e.g., *Xenia* sp. by Strychar et al. (2005) and other octocoral species in diverse scenarios (Prada, Weil & Yoshioka, 2010; Rossi et al., 2018; Slattery, Pankey & Lesser, 2019). However, bleaching was not observed during our experiments despite the decreased photosynthetic activity in *X. umbellata* under warmer temperatures. These observations align with the idea that autotrophy reliance does not appear to necessarily explain bleaching sensitivity in octocorals (Lasker et al., 2020). Moreover, the notion that soft corals might be less responsive to warm water bleaching than hard corals has been persistent (Prada, Weil & Yoshioka, 2010; Schubert, Brown & Rossi, 2016; Goulet et al., 2017; Lasker et al., 2020). Even though this is still uncertain given the lack of studies testing this specific hypothesis, it is clear that there is a degree of species-specific resistance concerning climate change impacts that will underpin the sets up for potential winners and losers in future scenarios.

Tolerance to environmental pressures in the long term should depend on the sustainability of energy allocation under stress, the long-term impact on organismal performance, and the ability to support other essential physiological processes (Pan, Applebaum & Manahan, 2015). Time significance in our study may support the idea that the longer the colonies remain under stress, the stronger its effects may become. This factor significance was supported for our single DOC addition experimental phase during the first 21 days. However, this could not be addressed for the second phase of our experiments (i.e., days 21 to 45), where we increased temperature together with continuous DOC addition. Further work is necessary to elucidate the effects of longer terms of stress in these corals and pathways of energy acquisition and allocation that may underpin tolerance mechanisms in these species when incubated under organic enriched conditions. However, the long-standing question in metabolic regulation remains at what point sublethal stress would become lethal due to energy limitations (Pan, Applebaum & Manahan, 2015).

## Conclusions

Overall, the present study shows that the photosynthesis and respiration of the pulsating xeniid *X. umbellata* is completely insensitive to high organic eutrophication concentrations between 10 and 40 mg L^−1^ at 26 °C and under heat stress over time. These findings provide evidence that the effects of the DOC stress factor alone are potentially trivial for this group of corals. In contrast, *X. umbellata* portrays negative sensitivity to temperature in terms of its photophysiology, which remained mild at intermediate thermal stress (i.e., 28 °C) and increased at higher temperatures, as observed at 30 °C and 32 °C. Nevertheless, bleaching was not observed, and at 32 °C, the colonies remained above compensation (P:R = 1.0) and below the net autotrophy threshold (P:R = 1.5), indicating mixotrophic behaviour, with displacement towards heterotrophy as temperature increased. These results may suggest that the effects of elevated temperatures on *X. umbellata* can be potentially modulated. Such modulation could occur via modifications of the total supply and demand for metabolic energy whenever possible (Comeau, Carpenter & Edmunds, 2017). However, it remains to be further investigated if they can be effectively overcome.

Interestingly, there was no statistical evidence for a synergistic interaction between DOC and temperature in the metrics assessed in our study. DOC organic eutrophication expected to worsen the effects of thermal stress in hard corals (Pogoreutz et al., 2017), did not show, from a physiological point of view, any statistical effects supporting DOC sensitivity enhancements towards increased temperature stress in *X. umbellata*; neither statistical effects supporting DOC enhanced thermal tolerance via utilization of DOC as an additional energetic resource (Bednarz et al., 2012; Vollstedt et al., 2020). Nevertheless, there is likely a potential additive or synergistic effect of DOC and heat stress through different mechanisms, which have yet to be confirmed.

Altogether our findings show that soft corals can be remarkably resistant to DOC effects, in contrast to hard corals that are susceptible to DOC. Under nutrient enrichment and highly eutrophicated environments, hard corals have shown pronounced negative responses such as (but not limited to), e.g., bleaching, increased mortality and affected photophysiology (Kuntz et al., 2005; Kline et al., 2006; Haas et al., 2016; Pogoreutz et al., 2017; Morris et al., 2019). Our findings show that *X. umbellata* soft corals can better withstand highly increased DOC conditions. Thus, portraying higher resistance to this stressor than hard corals. As a final remark, it would be valuable to visit further the conceptual framework within which organic eutrophication effects, together with thermal stress, are understood in soft corals (and for most octocoral species), as many particularities and the mechanics of how resistance may be achieved in soft corals remains unknown.

##  Supplemental Information

10.7717/peerj.11663/supp-1 Supplemental Information 1 Incubation measurements: single DOC addition, day 1 to 21

10.7717/peerj.11663/supp-2 Supplemental Information 2 Incubation measurements: DOC addition and simulated warming, day 21 to 45

10.7717/peerj.11663/supp-3 Supplemental Information 3 Survival counts and visual bleaching assessment during the experimental period

10.7717/peerj.11663/supp-4 Supplemental Information 4 Summary tables: Tukey pairwise comparisons of means for the temperature factor Tukey contrasts for gross and net photosynthesis, respiration rates and the P:R ratio of *X. umbellata* corals, under simulated warming and DOC additions.

10.7717/peerj.11663/supp-5 Supplemental Information 5 *P*-value plots: temperature factor pairwise comparisons Tukey adjusted *P*-value plots of estimated marginal means for gross and net photosynthesis, respiration rates and the P:R ratio of *X. umbellata* corals under simulated warming and DOC additions.

## Additional Information and Declarations

### Data Availability

The following information was supplied regarding data availability: The raw data measurements are available in the Supplemental Files.
