# Supplementary material for: Photosynthesis and respiration of the soft coral Xenia umbellata respond to warming but not to organic carbon eutrophication
Source: PeerJ. 2021 Jul 27;9:e11663. doi: 10.7717/peerj.11663 (PMC8323596; doi:10.7717/peerj.11663)
Supplement: Supplemental Information 4 — Tukey contrasts for gross and net photosynthesis, respiration rates and the P:R ratio of X. umbellata corals, under simulated warming and DOC additions. [file peerj-09-11663-s004.docx]

**Supplemental Information S1. Summary tables:** **Tukey pairwise comparisons of means for the temperature factor.** Tukey contrasts for gross and net photosynthesis, respiration rates and the P:R ratio of *X. umbellata* corals, under simulated warming and DOC additions.

**Table S1.1.** Temperature pairwise mean comparisons for *X. umbellata* gross photosynthesis. Tukey contrast P-values.

| **Contrast** | **Estimate** | **Std. Error** | **z value** | **p-value** |
| --- | --- | --- | --- | --- |
| 26 - 28 | -7.5817 | 1.1165 | -6.790 | **4.47e-11 ***** |
| 26 - 30 | -11.9203 | 1.1193 | -10.650 | **< 2e-16 ***** |
| 26 - 32 | -11.0462 | 1.1193 | -9.869 | **< 2e-16 ***** |
| 28 - 30 | -4.3385 | 0.7745 | -5.602 | **6.36e-08 ***** |
| 28 - 32 | -3.4645 | 0.7742 | -4.475 | **1.53e-05 ***** |
| 30 - 32 | 0.8740 | 0.7782 | 1.123 | 0.261 |

**Note:** *P*-values defined as significant at a threshold of P < 0.05 are highlighted in bold.

**Table S1.2.** Temperature pairwise mean comparisons for *X. umbellata* respiration. Tukey contrast P-values.

| **Contrast** | **Estimate** | **Std. Error** | **z value** | **p-value** |
| --- | --- | --- | --- | --- |
| 26 - 28 | -1.7566 | 0.6157 | -2.853 | **0.00433 **** |
| 26 - 30 | -4.7236 | 0.6189 | -7.632 | **1.39e-13 ***** |
| 26 - 32 | -3.2084 | 0.6157 | -5.211 | **7.50e-07 ***** |
| 28 - 30 | -2.9670 | 0.4295 | -6.908 | **2.45e-11 ***** |
| 28 - 32 | -1.4518 | 0.4247 | -3.418 | **0.00126 **** |
| 30 - 32 | 1.5152 | 0.4293 | 3.529 | **0.00125 **** |

**Note:** *P*-values defined as significant at a threshold of P < 0.05 are highlighted in bold.

**Table S1.3.** Temperature pairwise mean comparisons for *X. umbellata* net photosynthesis. Tukey contrast P-values.

| **Contrast** | **Estimate** | **Std. Error** | **z value** | **p-value** |
| --- | --- | --- | --- | --- |
| 26 - 28 | -5.4096 | 0.7017 | -7.709 | **5.06e-14 ***** |
| 26 - 30 | -7.5156 | 0.7035 | -10.683 | **< 2e-16 ***** |
| 26 - 32 | -7.8073 | 0.7035 | -11.098 | **< 2e-16 ***** |
| 28 - 30 | -2.1060 | 0.4868 | -4.326 | **3.04e-05 ***** |
| 28 - 32 | -2.3977 | 0.4868 | -4.925 | **2.53e-06 ***** |
| 30 - 32 | -0.2917 | 0.4894 | -0.596 | 0.551 |

**Note:** *P*-values defined as significant at a threshold of P < 0.05 are highlighted in bold.

**Table S1.4.** Temperature pairwise mean comparisons for *X. umbellata* P:R ratio. Tukey contrast P-values.

| **Contrast** | **Estimate** | **Std. Error** | **z value** | **p-value** |
| --- | --- | --- | --- | --- |
| 26 - 28 | -0.6651 | 0.1734 | -3.837 | **0.000499 ***** |
| 26 - 30 | -0.9063 | 0.1742 | -5.202 | **9.83e-07 ***** |
| 26 - 32 | -1.0305 | 0.1738 | -5.930 | **1.82e-08 ***** |
| 28 - 30 | -0.2412 | 0.1199 | -2.011 | 0.088577. |
| 28 - 32 | -0.3654 | 0.1193 | -3.063 | **0.006577 **** |
| 30 - 32 | -0.1242 | 0.1206 | -1.030 | 0.303036 |

**Note:** *P*-values defined as significant at a threshold of P < 0.05 are highlighted in bold.
