# Supplementary material for: Photosynthesis and respiration of the soft coral Xenia umbellata respond to warming but not to organic carbon eutrophication
Source: PeerJ. 2021 Jul 27;9:e11663. doi: 10.7717/peerj.11663 (PMC8323596; doi:10.7717/peerj.11663)
Supplement: Supplemental Information 5 — Tukey adjusted P-value plots of estimated marginal means for gross and net photosynthesis, respiration rates and the P:R ratio of X. umbellata corals under simulated warming and DOC additions. [file peerj-09-11663-s005.docx]

**Supplemental Information S2. Temperature factor pairwise comparisons.** Tukey adjusted P-value plots of estimated marginal means for gross and net photosynthesis, respiration rates and the P:R ratio of *X. umbellata* corals under simulated warming and DOC additions.

**Figure S2.1.** Temperature pairwise comparison for *X. umbellata* gross photosynthesis. Tukey adjusted P-value plot.

**Figure S2.2.** Temperature pairwise comparison for *X. umbellata* respiration. Tukey adjusted P-value plot.

**Figure S2.3.** Temperature pairwise comparison for *X. umbellata* net photosynthesis. Tukey adjusted P-value plot.

**Figure S2.4.** Temperature pairwise comparison for *X. umbellata* P:R ratio. Tukey adjusted P-value plot.
